# Using human-in-the-loop optimization for guiding manual prosthesis adjustments: a proof-of-concept study

**DOI:** 10.3389/frobt.2023.1183170

**Published:** 2023-07-19

**Authors:** Siena C. Senatore, Kota Z. Takahashi, Philippe Malcolm

**Affiliations:** ^1^ Biomechanics Research Building, University of Nebraska at Omaha, Omaha, NE, United States; ^2^ Department of Health and Kinesiology, University of Utah, Salt Lake City, UT, United States

**Keywords:** prosthesis fitting, patient-centered design, device optimization, prosthesis simulator, biomechanics

## Abstract

**Introduction:** Human-in-the-loop optimization algorithms have proven useful in optimizing complex interactive problems, such as the interaction between humans and robotic exoskeletons. Specifically, this methodology has been proven valid for reducing metabolic cost while wearing robotic exoskeletons. However, many prostheses and orthoses still consist of passive elements that require manual adjustments of settings.

**Methods:** In the present study, we investigated if human-in-the-loop algorithms could guide faster manual adjustments in a procedure similar to fitting a prosthesis. Eight healthy participants wore a prosthesis simulator and walked on a treadmill at 0.8 ms^−1^ under 16 combinations of shoe heel height and pylon height. A human-in-the-loop optimization algorithm was used to find an optimal combination for reducing the loading rate on the limb contralateral to the prosthesis simulator. To evaluate the performance of the optimization algorithm, we used a convergence criterium. We evaluated the accuracy by comparing it against the optimum from a full sweep of all combinations.

**Results:** In five out of the eight participants, the human-in-the-loop optimization reduced the time taken to find an optimal combination; however, in three participants, the human-in-the-loop optimization either converged by the last iteration or did not converge.

**Discussion:** Findings from this study show that the human-in-the-loop methodology could be helpful in tasks that require manually adjusting an assistive device, such as optimizing an unpowered prosthesis. However, further research is needed to achieve robust performance and evaluate applicability in persons with amputation wearing an actual prosthesis.

## 1 Introduction

Approximately one million adults in the United States live with a lower limb amputation (Ziegler-Graham et al., 2008). Individuals with amputation rely on a prosthesis to regain functionality in their lives. For this reason, significant research has focused on the design of passive (Collins et al., 2015; Etenzi et al., 2020) and active prostheses (Herr & Grabowski, 2012). While remarkable advancements have been made in prosthesis design, recent investigations suggest that individuals with amputation are more likely to develop osteoarthritis in their contralateral limb, despite being fitted with a state-of-the-art prosthesis (Ding et al., 2021). Individuals with amputation may experience decreased quality of life due to the increased risk of developing joint osteoarthritis in the knee of their contralateral limb (Burke et al., 1978; Lemaire & Fisher, 1994; Norvell et al., 2005; Struyf et al., 2009). During standing, weight-bearing for persons without amputation is presumed to be shared equally between lower limbs. However, it is believed that persons with amputation stand with greater sway and more weight-bearing towards their contralateral limb (Isakov et al., 1992; Rossi et al., 1995; Nadollek et al., 2002). Some studies suggest that increased time spent on the contralateral limb is an attempt to protect the soft tissues of the residual limb, which are not suited for weight-bearing immediately after amputation (Silver-Thorn et al., 1996). Regardless of the cause of gait deviation, the load placed on the contralateral limb is greater than the force that people without amputation exert on their lower limbs during natural locomotion (Suzuki, 1972; Engsberg et al., 1991; Engsberg et al., 1993). Consequently, this mechanism can put persons with amputation at a higher risk of developing osteoarthritis in their contralateral limb.

Previous studies investigated the effects of prosthetic components on the contralateral limb to explore the reason for gait deviation in persons with amputation. Studies have found that changing pylon flexibility can affect the vertical loading rate on the contralateral limb (Coleman et al., 2001). Additionally, socket fit and alignment are critical for appropriate function and comfort, as these factors are known to influence the contralateral limb loading rate (Zhang et al., 2019). Studies have suggested that the mechanics of prosthetic components may mitigate some compensatory mechanisms during locomotion in persons with amputation (Maun et al., 2021). With this, it is evident that a prosthetic device has many parameter settings that can be altered to achieve optimal comfort and fit.

During a fitting session, the settings of a prosthesis are adjusted to improve goals such as overall fit, satisfaction with the device, and characteristics of the walking gait pattern. Approximately 68%–88% of persons with amputation wear a prosthesis at least 7 h a day to aid in mobility and the performance of everyday activities (Pohjolainen et al., 1990; Walker et al., 1994; Jones et al., 1997). Despite the high rate of prosthesis use, there is a high rate of dissatisfaction with the comfort of prostheses (Dillingham et al., 2001; Pezzin et al., 2004). Several reasons could cause dissatisfaction with the comfort of the prosthesis. There can be errors in clinical measurements of the limb dimensions, partly due to difficulties locating the exact bony landmarks through layers of soft tissues. Additionally, errors can occur due to the prevalence of iliac asymmetries (Ingelmark & Lindstrom, 1963). Asking the individual for their opinion on their prosthetic may result in errors as their opinion is subjective, considering if their previous prosthetic fit was less than optimal (Friberg, 1984; Boone et al., 2012). From this, it is evident that the process of fitting a prosthesis can be improved. In addition, to appropriately fit a prosthesis the parameter settings of different prosthetic components, like pylon height and stiffness, need to be adjusted. Since different prosthetic components need to be altered and tested, this process can be time-consuming for both the patient and the prosthetist.

Advances in optimization algorithms have proven very useful in selecting optimal settings for exoskeletons (Zhang et al., 2017). Human-in-the-loop optimization algorithms, which optimize parameters while considering multiple interactions, have proven very useful in advancing the optimization of robotic exoskeletons (Malcolm et al., 2017; Zhang et al., 2017). Instead of analyzing measurements after completing a lengthy protocol of multiple parameter settings, these algorithms take measurements from a few parameter settings and converge in real time toward an optimal setting. These human-in-the-loop algorithms have been used to optimize devices in response to the user’s physiological changes (i.e., metabolic cost) (Koller et al., 2016). This methodology takes inspiration from humans who naturally optimize their coordination patterns for energy cost and other aspects of locomotor performance (Alexander McN., 1989; Selinger et al., 2015). Studies have demonstrated that human-in-the-loop optimization can improve the performance of wearable devices like robotic exoskeletons (Felt et al., 2015; Koller et al., 2016; Zhang et al., 2017; Ding et al., 2018). In addition, it is known that human-in-the-loop optimization algorithms emphasize the importance of customization and individualism in assistive devices (Koller et al., 2016; Zhang et al., 2017). However, human-in-the-loop optimization has yet to be used to guide manual adjustments for optimizing prostheses.

The goal of this study was to evaluate the usability of human-in-the-loop optimization in prescribing manual adjustments of shoe heel height and pylon height to reduce the loading rate on the contralateral limb. Our first aim was to evaluate the time required to find the optimal parameter combination using human-in-the-loop optimization. The algorithm was designed to simultaneously optimize shoe heel height on the contralateral limb and pylon height on the prosthesis simulator limb as a means of converging to a parameter combination that minimizes the loading rate on the contralateral limb. We chose to alter shoe heel height on the contralateral side as previous studies have shown that shoe heel height can affect knee joint loading (Shakoor et al., 2010). In addition, we chose pylon height since it was the most feasible component to alter for this preliminary study and is known to affect the fit and alignment of a prosthesis. We hypothesized that the algorithm would reduce the time necessary to reach a minimal loading rate compared to the time required to complete a sweep of all the possible parameter combinations. Our second aim was to analyze the accuracy of the human-in-the-loop optimization algorithm in finding an optimal combination. By comparing the loading rate on the contralateral limb from the sweep and optimization methods, we evaluated the accuracy of the human-in-the-loop optimization algorithm. Since persons with amputation are such a diverse population, implementing this methodology could accommodate more specific customization during fitting processes and allow a prosthesis to achieve its potential.

## 2 Materials and methods

### 2.1 Subject recruitment

As a preliminary step towards testing in persons with an amputation, ten healthy young adults (*n* = 10; mass, 76.4 ± 15.5 kg; height, 1.73 ± 0.08 m; mean ± SD) were recruited. The goal of this study was not to obtain representative normative data of the average person with an amputation; instead, the goal was to evaluate the efficiency of the optimization. Because of this specific goal, we believed a relatively small sample and a convenience sampling strategy was acceptable (Kim et al., 2020). All participants were recruited within the Biomechanics Research Building at the University of Nebraska at Omaha. All recruited participants were able to provide informed consent. The study was approved by the University of Nebraska Institutional Review Board.

**FIGURE 1:**
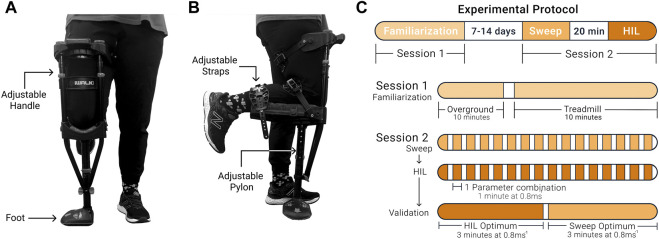
The prosthesis simulator and experimental protocol. **(A)** Front view. The foot’s orientation on the device could be switched depending on whether the participant was left or right-footed. **(B)** Side view. The prosthesis simulator had three straps. The straps secured the lower leg to the device to prevent the participant from using their lower leg and could be easily tightened or loosened. The lower portion of the device was raised and lowered to change the pylon height parameter setting. **(C)** Protocol timeline.

A health questionnaire was administered to assess if the participant had any functional limitations that impacted their capacity to complete the protocol. We based the inclusion criteria on the subject’s age, height, and leg length. We only included participants between 19–45 years old. In addition, we only included participants who could fit the prosthesis simulator using the manufacturer’s leg length and height restriction (iWALK 2.0, Long Beach, CA, United States, Figures 1A, B). We only included participants free of conditions limiting walking capability, including joint, musculoskeletal, or neurological issues. Additionally, we only included participants who were free of any cardiovascular pathologies.

### 2.2 Experimental protocol

Participants walked with a device that simulated walking with a prosthesis (Figures 1A, B). This device and similar devices have been used in various studies to simulate walking with a prosthesis (Keeken et al., 2012; Ramakrishnan et al., 2017; Schlafly & Reed, 2020a). Anecdotally, we can report that none of the participants had prior experience with the prosthesis simulator or similar devices. Participants completed two sessions (Figure 1C). The initial session was a familiarization session to mitigate potential learning effects during the testing session. During this session, participants walked with the prosthesis simulator on the neutral setting (no shoe heel height and the initial fitted pylon height; combination 1, 3) to represent walking with a device that has not been adjusted. The prosthesis simulator was used on the participant’s dominant limb, which was determined based on which leg they would use to kick a ball (van Melick et al., 2017). Participants walked overground and then progressed onto the treadmill for 20 min, where the speed increased until 0.8 ms^−1^ was achieved. During the second session, participants completed three experimental protocols: a parameter setting sweep protocol where all conditions were tested (sweep), followed by the human-in-the-loop optimization protocol (HIL optimization), and finally, a validation test of the optimal combinations determined from the sweep and the optimization protocols. During all experimental protocols, the participants walked at 0.8 ms^−1^. Studies using similar simulator devices used a similar, relatively low walking speed (Vanicek et al., 2007; Schlafly & Reed, 2020b). On average, we paused about 2 min between conditions to calculate loading rates, change the settings and let participants rest. Participants were free to rest longer for up to 5 min. Anecdotally, participants did report minor fatigue due to walking with the prosthesis simulator toward the end of the protocol.

### 2.3 HIL optimization protocol

Participants walked on the treadmill while wearing the prosthesis simulator for 1 min for each parameter combination. After completing each combination, the human-in-the-loop optimization algorithm prescribed the following combination to be evaluated. We manually changed the parameters to the combination that the algorithm prescribed. These adjustments were limited by the intervals between the physically available settings; therefore, the prescribed settings had to be rounded to the available setting intervals. Combinations were changed until 16 combinations were completed. The optimal combination determined from this protocol (i.e., the optimal determined by HIL optimization) was denoted as the HIL optimization optimum.

**FIGURE 2:**
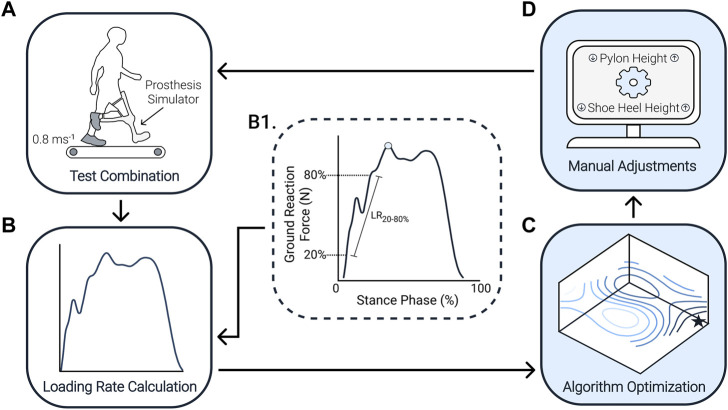
Human-in-the-loop optimization algorithm flowchart. **(A)** Participants walked on a treadmill at 0.8 ms^−1^ with the prosthesis simulator for each combination. **(B)** The treadmill recorded the ground reaction force. **(B1)** The loading rate was calculated from the ground reaction force by calculating the slope between 20%–80% from the first peak (blue circle). **(C)** We used gradient descent and successive parabolic optimization to find the optimal combination of shoe heel height and pylon height. **(D)** From this, the algorithm prescribes the following combination to test, that is, a specific shoe heel height and pylon height. This process continued until 16 combinations were completed. From those 16 combinations, we then determine the minimum amount that would have been required to converge on the optimum [**(C)**, black star] after the experiment. Often the human-in-the-loop algorithm repeats certain conditions rather than testing each of the 16 possible combinations like the sweep protocol.

We designed a human-in-the-loop optimization algorithm to minimize the loading rate on the limb contralateral to the prosthesis simulator (Figure 2; Supplementary Information S1). The loading rate was determined by calculating the vertical instantaneous loading rate from the ground reaction force (GRF) recorded at a frequency of 2000 Hz using an instrumented split-belt treadmill (Bertec, Columbus, OH, United States). The vertical instantaneous loading rate is preferable to the vertical average loading rate as it provides a more consistent evaluation (Ueda et al., 2016). We calculated the loading rate as the maximum of the instantaneous slope between 20%–80% from the first peak (Figure 2B1). This calculation method has been used in previous studies to calculate the loading rate (Abolins et al., 2019).

The algorithm uses gradient descent to guide the first parameter combinations and then uses successive parabolic optimization once a sufficient number of parameter combinations have been tested. These techniques are based on similar techniques adapted from previous studies (Koller et al., 2016; Molderez et al., 2017), where the goal was to find the local minimum of an objective function, similar to a ball rolling toward the lowest point of a valley. After testing the first combination of shoe heel height and pylon height, two neighboring combinations within the grid of all possible shoe heel height and pylon height combinations were randomly chosen to test. In order to perform a first estimation of the gradient in the three-dimensional space of shoe heel height and pylon height against loading rate, we needed to complete these three parameter combinations. This gradient was then used to calculate the direction of the estimated new optimal parameter combination. In this estimation, a set of hyper-parameters defined how far the new estimated optimum will be placed in the direction of the gradient.

Once four combinations were completed, we started using a successive parabolic optimization to update the algorithm’s estimate of the optimal parameter combination. At this point, we fit a paraboloid through all completed combinations. Given the small range over which the two parameters were adjusted, we assumed there should be only one optimal combination. If the parabolic fit was concave and pointed to a single optimum, we used the parabolic fit to define the new estimated optimum. If the paraboloid fit produced a non-concave surface (i.e., a surface that descends in many directions), the optimization process reverted to a gradient descent search instead of parabolic optimization. For the remainder of the combinations, we kept evaluating the parabolic fit and, when needed, the gradient descent search until 16 combinations were completed. Throughout the optimization process, it is possible that some combinations could be repeated.

In the initial stages of developing the optimization algorithm, we compared the suitability of three different optimization algorithms (the covariance matrix adaptation evolution strategy (CMA-ES) (Zhang et al., 2017; Ren et al., 2019), gradient descent (Felt et al., 2015), and successive parabolic optimization. We used simulated contralateral limb loading rate data obtained by generating previously measured contralateral limb loading rates with some added random noise from one participant (Supplementary Information S2). In this simulation study, we found that successive parabolic optimization was relatively more suitable for this application than the other optimization methods (Supplementary Information S3). We are uncertain why the present method performed slightly better. This may be associated with the type of simulated data generated for this comparison. Furthermore, specifics of the problem are relatively uncommon such as the very low resolution of only a 4 × 4 grid of possible combinations. It is also possible that this affected the outcome.

**FIGURE 3:**
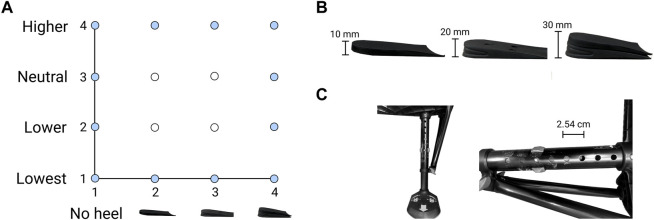
The parameter settings. Shoe heel height and pylon height were the two-parameter settings adjusted throughout the protocol. **(A)** Randomized initial combinations. The possible combination choices for the initial combination (dark circles) used in the human-in-the-loop optimization. These were randomized for each participant. **(B)** Shoe heel heights. Shoe heel heights were added to the shoe of the contralateral limb and included 10, 20, and 30 mm heights (left to right). The no-heel parameter setting indicated that no heel was added to the shoe. **(C)** Pylon heights. Pylon heights were adjusted on the prosthesis simulator and varied from two lower and one higher than the initial fitted height. Pylon height options differed by 2.54 cm.

The initial combination was randomly chosen for each participant. Similar to previous human-in-the-loop studies (Felt et al., 2015; Zhang et al., 2017; Ding et al., 2018; Ren et al., 2019), we restricted the initial combination to the combinations along the edge of the grid of all possible shoe heel height and pylon height combinations (Figure 3A). Since we assumed that the optimum most likely exists somewhere in the middle of the parameter combination grid, this restriction allows us to see how the algorithm converges to an optimum. Suppose the optimization process was to begin in the middle of the parameter combination grid; in that case, it may not be easy to distinguish whether the algorithm identifies the optimum or is not making any updates.

### 2.4 Sweep protocol

Participants walked on the treadmill while wearing the prosthesis simulator for 1 min for each shoe heel height and pylon height combination. Shoe heel heights were inserted in the shoe on the contralateral side and included 0, 10, 20, and 30 mm heights (Figure 3B), where 0 indicated no additional heel was inserted in the shoe. Pylon height was changed on the prosthesis simulator and ranged from one higher to two lower than the initial fitted height, where each setting differed by 2.54 cm (Figure 3C). We used a number code to designate each parameter setting: shoe heel heights of 0, 10, 20, and 30 mm were labeled as heel heights #1, 2, 3, and 4, respectively; pylon heights two lower and one higher than the initial fitted height were labeled as pylon heights #1, 2, 3, and 4, respectively where #3 was the initial fitted height. All 16 possible parameter combinations were completed in random order for each participant. Participants were allocated up to 5 min of rest between testing parameter combinations. The optimal combination determined from this protocol (i.e., the optimal from a 2D surface fitted through all 16 combinations) was denoted as the sweep optimum.

### 2.5 Validation tests

In addition, after completing the sweep and the optimization protocols, participants walked on the treadmill under the optimized parameter combination from the HIL protocol, followed by the optimal combination of the sweep protocol for 3 min each. Conducting this validation test allowed us to compare the contralateral limb loading rate between both optimized combinations. We used the neutral combination from the sweep protocol to compare the results to a device that is not individually adjusted at all. We repeated the optimum from both protocols because the optimal combination determined by the HIL optimization and sweep protocol might have had a low loading rate due to chance.

### 2.6 Statistical analysis

To find the optimal parameter combination from the sweep protocol, we fit a second-order polynomial that was a function of shoe heel height and pylon height against the loading rate. The minimum loading rate of this fitted surface determined the individual optimal combination. We reported the optimal parameter combination on a group level using the mean ± standard deviation.

We used a convergence criterium to evaluate the algorithm’s performance and determine when an optimal combination had been achieved in the HIL optimization protocol. Previous studies have used a similar convergence criterium as a performance metric for human-in-the-loop optimization algorithms (Felt et al., 2015; Zhang et al., 2017; Ding et al., 2018). An optimal combination was said to be achieved when prescribed combinations remained between the parameter setting one above and one below the estimated optimal parameter setting (Supplementary Information S4). The number of combinations it takes before staying within this band was defined as “combinations-to-convergence.” We reported the average number of combinations until convergence occurred based on the mean ± standard deviation. To evaluate if the number of combinations when convergence occurs was significantly smaller than the maximum number of combinations (i.e., 16), we used a one-sample t-test.

We also evaluated if there was a significant difference in parameter settings between the HIL optimization optimum and the sweep optimum using a paired t-test. To compare the average loading rate across participants for both optimal parameter combinations, we used a paired t-test with a Holm-Šidák correction. Additionally, we used a paired t-test to compare the optimal parameter combinations to the neutral combination to see if there were any significant changes in the loading rate on the contralateral limb compared to wearing a device that is not individually adjusted at all.

## 3 Results

Data analysis included eight of the ten recruited participants (*n* = 8). Data from two participants were excluded due to a problem with the zeroing of the force treadmill and an error in the sequence of conditions in the protocol.

### 3.1 Combinations-to-convergence

**FIGURE 4:**
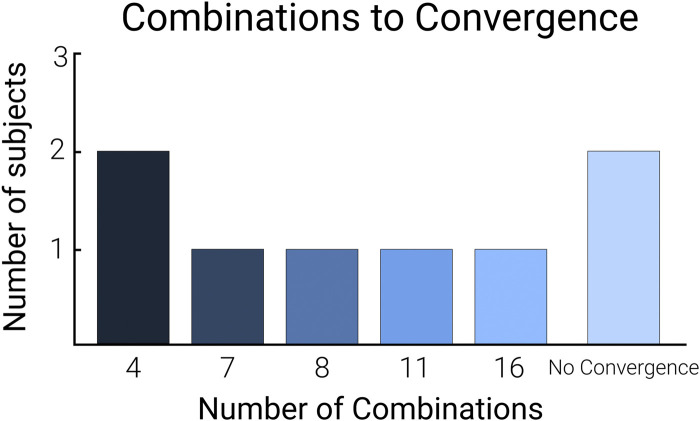
Combinations-to-convergence bar graph. The calculated combinations-to-convergence using the number of conditions tested to achieve the optimal combination. The no convergence bar represents the participants whose optimization protocol did not converge to an optimal combination.

The combinations-to-convergence was highly variable among participants (Figure 4; Supplementary Information S5.1). Half of the participants achieved the optimal in eight or fewer combinations. Two participants achieved the optimal in more than eight combinations, and two did not achieve an optimal combination (i.e., the prescribed optimum never stayed within the defined convergence band). The average combinations-to-convergence among the participants who did converge was 8.3 ± 4.6 combinations (mean ± standard deviation, *n* = 6). The two individuals who did not converge were excluded from this mean and standard deviation as they did not have a defined convergence. On average, the time taken for the human-in-the-loop optimization algorithm to achieve the optimum was significantly lower than completing the total number of combinations (*p* < 0.05, *n* = 6).

### 3.2 Validation of optimal combinations

**FIGURE 5:**
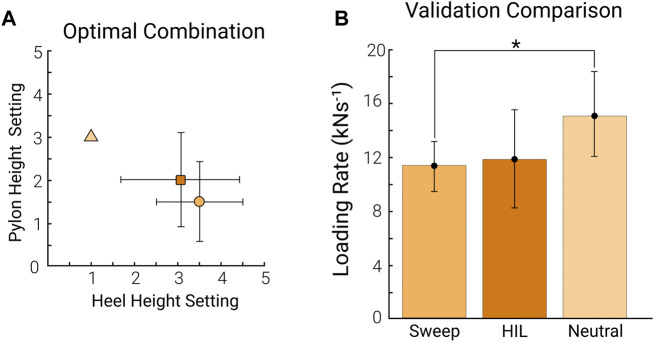
Optimal combination validation. The comparison between the optimal determined by the *sweep* (orange) and the optimal achieved by *HIL optimization* (dark orange) **(A)** The average of the optimal combination across participants from the *sweep* (orange circle) compared to the optimal combination across participants for the *HIL optimization* (dark orange square). The neutral combination is denoted as the light orange triangle for reference. The error bars represent the standard deviation across participants (*n* = 8). **(B)** The average loading rate across participants from the optimal combination from the *sweep,* the optimal combination from the *HIL optimization* (HIL), and the neutral combination (combination 1, 3). The error bars represent the standard deviation across participants (*n* = 8).

The average optimal combination determined by the *sweep* was parameter setting 3.5 ± 1.0 and 1.5 ± 0.9 for shoe heel height and pylon height, respectively (mean ± standard deviation, *n* = 8). The average of the optimal combination determined by the *HIL optimization* was parameter setting 3.1 ± 1.4 and 2.0 ± 1.1 for shoe heel height and pylon height, respectively. There was no significant difference in the parameter settings between the *sweep* and *HIL optimization* optimum (*p* = 0.785 for shoe heel height, *p* = 0.275 for pylon height; Figure 5A; Supplementary Information S5.2). In the validation tests, we used rounded approximations of the optimal parameter combination from each protocol since we could only test available settings. The average from the tested combinations during the validation tests for the optimum of the *sweep* was 3.4 ± 1.0 and 1.5 ± 0.8 for shoe heel height and pylon height, respectively. The average optimal parameter combination for the validation of the *HIL optimization* was 3.1 ± 1.6 and 2.0 ± 1.0 for shoe heel height and pylon height, respectively.

The average loading rate from the *sweep* optimum was 11.5 ± 1.7 kN s^−1^. The average loading rate from the *HIL optimization* optimum was 11.9 ± 3.6 kN s^−1^. The loading rate in the neutral combination setting (combination 1, 3) was 15.1 ± 3.3 kN s^−1^. There was no significant difference in the loading rate between the two optimal combinations (*p* = 0.730; Figure 5B). The *sweep* optimum and the *HIL optimization* optimum reduced the loading rate by 23.3% and 20.7%, respectively, compared to the neutral combination. The *sweep* optimum had a significantly lower loading rate than the neutral combination (*p* < 0.05). However, there was no significant difference in loading rate between the *HIL optimization* optimum and the neutral combination (*p* = 0.169).

Since the HIL protocol did not show convergence in all participants, we conducted a follow-up test. We used a paired t-test to compare the average loading rate across participants who converged to an optimal combination (*n* = 6). When considering only the participants who did converge, both optimums from the *sweep* and *HIL optimization* had significantly lower loading rates than the neutral combination (*p* < 0.05, Supplementary Information S5.3).

## 4 Discussion

This study investigated if a human-in-the-loop optimization algorithm can guide manual adjustments to optimize a prosthesis simulator. We hypothesized that the human-in-the-loop optimization algorithm would reduce the time taken to find an optimal parameter setting. The findings show that the human-in-the-loop optimization algorithm reduced the time taken to find an optimal combination in 5 out of 8 participants, partially accepting our hypothesis.

The human-in-the-loop optimization algorithm determined an optimal combination similar to the optimum determined by the sweep of all 16 combinations. However, a statistical power analysis shows that we have yet to determine whether this means that there is genuinely no difference or if this was due to the sample size, given that the statistical power was 0.375 and 0.289 for shoe heel height and pylon height, respectively. The loading rate for both optimal combinations was similar, further validating that the human-in-the-loop optimization could reduce the loading rate similar to the sweep protocol. However, the fact that the algorithm did not converge in one-fourth of the participants raises concerns about the robustness of the optimization algorithm. While this seems to question the robustness of the optimization algorithm, previous studies show that this is not an uncommon result (Zhang et al., 2017; Welker et al., 2021). A particular study stated that none of their optimization algorithms could reduce metabolic cost significantly (Welker et al., 2021). Additionally, a different study mentioned instances where researchers had to reset the algorithm and add additional walking time (Zhang et al., 2017). On the contrary, supplementary analysis of the variability between repetitions of the same condition may suggest that the chosen optimization problem was simply very challenging (Supplementary Information S6). We also investigated whether any of the features of the algorithm, such as the frequency of switching between parabolic optimization and gradient descent, was related to the time-to-convergence performance. Still, we did not find any clear relationship there.

**FIGURE 6:**
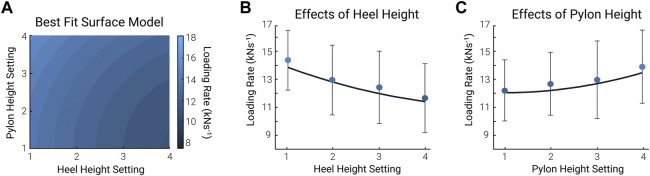
Linear mixed-effect model. We used a 2nd order polynomial as the best-fit model to analyze the effect of shoe heel height and pylon height on the contralateral limb loading rate. **(A)** The surface plot of the linear best-fit model. The pylon height setting is on the vertical axis, and the shoe heel height setting is on the horizontal. The color bar represents the loading rate, where light blue is the highest and dark blue is the lowest. **(B,C)** 2-Dimensional plot. The effect of shoe heel height **(B)** and pylon height **(C)** on the contralateral limb loading rate. This 2-dimensional plot was taken from the middle point of pylon height and shoe heel height from **(A)**, the mean of conditions 2 and 3. The circles and error bars in **(B)** represent the mean ± standard deviation of all pylon heights at each shoe heel height setting. The circles and error bars in **(C)** represent the mean ± standard deviation of all shoe heel heights at each pylon height setting (*n* = 8).

Although using the human-in-the-loop optimization algorithm reduced the time to find an optimal combination for over half of the participants, one participant required all 16 combinations to find an optimal combination. Additionally, the algorithm never converged to an optimal combination for two of the participants. This finding raised the question of whether this variability in the effectiveness was due to the algorithm or rather the effects of the prescribed parameter combination being small or inconsistent. To investigate this question, we performed a supplementary analysis of the statistical significance of the effects of shoe heel height and pylon height on loading rate based on the data from the sweep protocol. We used the following linear mixed-effect model (1) to study the effects of shoe heel height and pylon height on the loading rate on the contralateral limb:

$$z_{Fit}=c_{1}x^{2}+c_{2}x+c_{3}y^{2}+c_{4}y+c_{5}$$ (1)

where x, y, and z are shoe heel height, pylon height, and loading rate, respectively, terms $c_{1}$ to $c_{4}$ are the coefficients for each independent parameter setting, and $c_{5}$ is the constant intercept term. We found no statistical significance for each of the terms (*p*-values were 0.629, 0.775, 0.243, and 0.383 for shoe heel height, the square of shoe heel height, pylon height, and the square of pylon height, respectively; Figure 6). On the one hand, this means that the effects of each parameter setting were inconsistent across all participants. This suggests that the effects of the parameters were relatively small and not highly repeatable. Anecdotally, we can comment that the ranges in shoe heel height and pylon height were sufficiently large to make walking difficult at the extreme ends of the parameter settings (e.g., walking with the greatest shoe heel height or pylon height). Because of this, it is unlikely that the lack of statistically consistent effects is likely not due to having chosen too small of a range. This lack of statistically significant consistency in the effects of the independent parameters could explain why the optimization protocol did not converge for all participants. On the one hand, this may emphasize that the effect of the parameter settings was variable across participants, highlighting the need for a unique optimization method like human-in-the-loop optimization to find each individual’s optimum. On the other hand, this may also suggest that the selected parameter settings may not have been the most relevant settings to optimize. Other studies sometimes also acknowledge that other parameter settings that may be more sensitive to the cost function could have been selected (Peng et al., 2022). Future investigations should optimize different parameter settings that have been shown to affect the contralateral limb, like pylon flexibility (Coleman et al., 2001) and stiffness (Maun et al., 2021).

**FIGURE 7:**
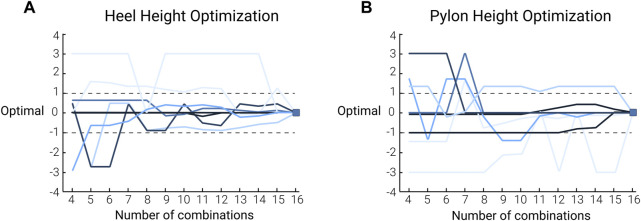
Human-in-the-loop optimization histories of participants: the pattern of shoe heel height **(A)** and pylon height **(B)** optimization during the *HIL optimization* for each participant. The colors of the lines relate to the convergence metric where the dark blue lines represent participants who converged in 4 combinations, and the light blue lines represent participants who did not converge to an optimal combination. The optimization history pattern is plotted relative to the final optimal parameter setting determined by the *HIL optimization* to visually see the convergence. As such, each line ends at 0 on the vertical axis. The dashed lines represent the band that was used to determine whether the algorithm achieved convergence or not. More specifically, we considered the algorithm to have converged if the prescribed parameter combination stayed within a band of ± 1 (*n* = 8).

While previous studies have proven the effectiveness of human-in-the-loop optimization in tuning one or multiple parameters, the application of this methodology for optimizing manual adjustments of assistive devices is novel. Upon further analysis, it appears that the algorithm could optimize both parameter settings in some participants, while in others it only optimized one or neither. Figure 7 is a visual representation showing the variability of the optimization patterns for both parameter settings. This emphasizes that while the parameter settings together did not affect the loading rate on the contralateral limb, there is potential for this methodology to guide manual adjustments. Specifically, it illustrates that the optimal shoe heel height (Figure 7A) was achieved more efficiently and consistently across participants than the pylon height (Figure 7B). Footwear parameters on the contralateral limb are not typically modified in persons with amputation. However, this finding suggests that further analyses into the importance of footwear parameters on the loading rate on the contralateral limb in persons with amputation may be beneficial. Additionally, further investigations should be done to validate the use of improved human-in-the-loop optimization algorithms for simultaneously optimizing two manually adjusted parameters.

There are some limitations to this study. Participants were recruited for this experiment through convenience sampling on a college campus. Although the recruitment age ranges from 19 to 45, the sample may only represent part of the population. Concerning the protocol, not all parts of the experiment were randomized. It is possible that some of the differences between the sweep and HIL optimum could be due to adaptation or fatigue. However, we think the habituation was sufficient since the purpose of the study was to compare the efficiency of the optimization algorithm. While similar prosthesis simulators have been used to simulate walking with a prosthesis, the findings from this study likely do not reflect persons with amputation. To validate the results of this study, the protocol could be implemented as a case study on a person with an amputation. With this, it could be possible that the optimization algorithm could improve as persons with an amputation who have experience walking with a prosthesis could have a more consistent gait pattern. It is known that persons with amputation have and need much more time to be able to get used to walking with a prosthesis (Barr et al., 2012; Ray et al., 2018), increasing the chance for a more consistent gait pattern. This higher consistency has the potential to make the optimization process more straightforward.

Persons with amputation lack both sensing and direct control of the mechanics of their prosthetic foot and ankle (Welker et al., 2021). With this, the sensory feedback must come from the interactions at the socket and whole-body proprioception (Welker et al., 2021). The importance of sensory feedback reiterates why human-in-the-loop optimization is successful with exoskeletons and might be harder to replicate in devices such as prostheses. It may be hard to implement human-in-the-loop optimization in persons with amputation as the contributions to differences in gait go deeper than just the effects of component mechanics (Welker et al., 2021). Investigations to validate the implementation of human-in-the-loop optimization in persons with amputation should consider different cost functions other than metabolic cost to optimize the prosthesis. Since previous studies have reported that prosthetic components affect peak ground reaction force (Grabowski & D’Andrea, 2013; Morgenroth et al., 2011) and knee external adduction moment (Grabowski & D’Andrea, 2013; Morgenroth et al., 2011), future research could investigate optimizing these variables using human-in-the-loop optimization. Regarding the parameter settings selected to adjust, there are some limitations in clinical applicability in persons with amputation, as prosthetists traditionally do not alter the contralateral limb. The results from the linear mixed effect model further reiterate the limitations in the effectiveness of altering the selected parameter settings. In addition, the shoe heel height stiffness was not considered, although it is evident that stiffness influences limb loading (Hong et al., 2013; Kulmala et al., 2018). Future investigations could analyze the implementation of human-in-the-loop optimization in optimizing applicable clinical parameters like pylon height and heel height stiffness on the prosthesis side.

Another limitation is that the algorithm was used to optimize parameters that only have 4 settings. On the one hand, it is possible that the actual optimum in certain participants would have existed outside of the range of the tested combinations. On the other hand, the small number of settings may have favored the sweep protocol considering all possible combinations were tested. It is possible that optimizations with a greater resolution of options may have resulted in a more favorable result; however, there is no evidence that this would have been better. Further investigations are needed to evaluate the effect of a greater parameter setting resolution in human-in-the-loop optimization of manually adjusted devices. To minimize the chances of the initial combination being optimal, we restricted the initial combination to the combinations along the border of the available choices. However, in some instances, the initial combination that was tested turned out to be close to the final optimum. It is possible that those participants would have produced a different result that showed convergence if their protocol started out from a combination that was further from the optimum. Finally, we only considered one possible algorithm that included gradient descent and successive parabolic optimization techniques. Further investigations could investigate methods like Bayesian optimization (Brochu et al., 2010; Kim et al., 2017; 2019) or covariance matrix adaption evolution strategy CMA-ES (Zhang et al., 2017; Ren et al., 2019).

## 5 Conclusion

The study implemented a human-in-the-loop optimization algorithm to guide manual adjustments to optimize a prosthetic simulator. The findings from this study show that even though there is potential for this methodology to be implemented in the patient population of persons with amputation, many factors need to be considered. Since prosthetic components are known to affect contralateral limb loading, optimizing parameters on the prosthesis itself is a more clinically applicable approach to implementing this methodology in persons with amputation. Since persons with amputation rely on sensory feedback from the prosthesis, optimizing a cost function that is not related to physiological changes may be more beneficial in persons with amputation. Considering prosthetists typically look at both limbs when fitting and adjusting a prosthesis, future investigations could include a multi-objective optimization to examine the effects of changing multiple parameter settings on both limbs.

## Data Availability

The data supporting the conclusion of this article are available in Supplementary Material.
