# Supplementary material for: Using human-in-the-loop optimization for guiding manual prosthesis adjustments: a proof-of-concept study
Source: Front Robot AI. 2023 Jul 19;10:1183170. doi: 10.3389/frobt.2023.1183170 (PMC10394618; doi:10.3389/frobt.2023.1183170)
Supplement: Supplementary file 1 [file DataSheet1.pdf]

Supplementary Material

1 Optimization Algorithm Design

1.1 Algorithm Inputs

A processing algorithm in MATLAB (MathWorks Inc.) calculated the loading rate using the ground reaction force (GRF) recorded at a frequency of 2000 Hz using an instrumented split-belt treadmill (Bertec, Columbus, OH, USA) during each combination. We ran the GRF through a custom MATLAB script that stride normalized the GRF data and then filtered the GRF using a fourth-order low-pass Butterworth filter with a 20 Hz cut-off frequency. The algorithm calculated the loading rate using the vertical instantaneous loading rate. We calculated the loading rate as the slope of the GRF from 20% of the first peak until 80% of the first peak. The calculated loading rate and the associated shoe heel height and pylon height were fed as inputs into the human-in-the-loop optimization algorithm.

1.2 Gradient Descent

Since we need two points to be able to calculate the gradient, we needed to evaluate the settings near the estimated center location using the following equation:

$$\mathbf{Guess_{x,y} = Estimate_{x,y} - \frac{1 * Interval_{x,y}}{2}} \tag{2}$$

where $\text{Interval}_{x,y}$ determined how far from the estimate we wanted the guess to be. The $\text{Interval}_{x,y}$ was set at 1.5 and 1.1 for shoe heel height and pylon height, respectively. These values were selected based on a preliminary simulation study. This equation prescribed the following combination to test: a specific shoe heel height and pylon height. This algorithm was run until three combinations were completed.

After the initial three combinations had been tested, the human-in-the-loop optimization algorithm used a gradient descent search method to determine the following combination to be tested. The gradient is the slope of a function that allows us to predict the effect of various inputs. If the gradient is high, the function's slope will be steep, resulting in a faster learning speed. On the contrary, if the slope is zero, the learning will stop since a minimum of the function has been achieved. Ultimately, this would mean that an optimum has been achieved. By calculating the gradient, we evaluated which direction the function should head in. For this study, we evaluated the direction of the gradient for the last set of combinations. Since we had two parameters, we calculated the gradient in both the x and y direction: shoe heel height, and pylon height, respectively. We calculated the gradient of the last set of combinations tested in each direction using the following equation:

$$\mathbf{Gradient_{x,y} = \frac{z(end) - z(end-1)}{x,y(end) - x,y(end-1)}} \tag{3}$$

where x was the shoe heel height setting, y was the pylon height setting, and z was the associated loading rate.

Using the gradient of the combinations tested drove the parameter settings to a local minimum by associating the gradient of the last set of combinations tested with the parameter settings tested. We used a scheduled gain (hyper-parameters) to help estimate the gradient where their value decreased as the number of iterations increased (Felt et al., 2015). These hyper-parameters were designed to slightly improve the gradient descent functionality (Felt et al., 2015). By allowing us to choose how much the parameters are varied as a function of the gradient, we can make the variation smaller and smaller over time. The algorithm estimated the center location of the following combination to be tested around which the following gradient was estimated using the following equation:

$$\mathbf{Estimate_{x,y} = Mean\big(x, y(end - 1{:}\, end)\big) - \frac{\alpha_0 * Gradient * A_0}{(A_0 + i^{\gamma})}} \quad \textbf{(4)}$$

where α_0, A_0, and γ were the hyper-parameters that helped train the human-in-the-loop optimization algorithm.

In our experiment, we defined α_0, A_0, and γ for the shoe heel height and pylon height estimations. For shoe heel height, we set α_0, A_0, and γ at 1.8, 5.2, and 0.5, respectively. Additionally, for pylon height, we set α_0, A_0, and γ at 1.1, 3.1, and 1.0, respectively. These values were selected based on a preliminary simulation study.

1.3 Successive Parabolic Optimization

Once three combinations were completed, the following combinations were prescribed using a successive parabolic optimization. While gradient descent estimates the direction the function should head in, it does not estimate exactly where the optimum will be. A successive parabolic optimization is another technique used to find the minimum of a function. At this point, we fitted a paraboloid through the already completed combinations. Given the small range over which the two parameters were adjusted, we assumed there should be only one optimal combination. If the paraboloid function produced a non-U-shaped surface, the optimization process reverted to a gradient descent method. This is because the optimization would be restricted if the optimum were on the border of our possible combinations. However, if the paraboloid function produced a concave surface (i.e., presented a single optimal combination), the optimization process would continue using the paraboloid function. *It should be noted that this evaluation was done independently for the x and y directions. For example, if the algorithm produced a concave surface in the x-direction but a non-U-shaped surface in the y-direction, successive parabolic optimization was used to determine the next x parameter setting, while gradient descent was used to determine the next y parameter setting.* The optimization algorithm used the following paraboloid function *for the successive parabolic optimization*:

$$\mathbf{z_{\mathit{Fit}} = c_1x^2 + c_2x + c_3y^2 + c_4y + c_5} \quad \textbf{(5)}$$

where x, y, and z are shoe heel height, pylon height, and loading rate, respectively, c_5 is the constant intercept term, and c_1 to c_4 are the coefficients for each independent parameter setting. By updating this paraboloid fit as combinations are completed, the algorithm can also update its estimate of the optimal combination. The algorithm would prescribe the following combination to test by using the following equation:

$$\mathbf{NextGuess_{x,y} \approx \frac{-c_{2,4}}{2c_{1,3}}} \quad \textbf{(6)}$$

where x and y are shoe heel height and pylon height, respectively, $c_{2,4}$ is the coefficient for the first-order term for x and y, respectively, and $c_{1,3}$ is the coefficient for the second-order term for x and y, respectively. Being a paraboloid, the minimum of the function will be the optimal combination achieved by the algorithm for that participant. We expect that the optimal combination will have the lowest loading rate across all the possible combinations.

2 Preliminary Optimization Algorithm Development Using Simulated Data

During the development stages of this study, we pilot-tested algorithms based on preliminary data. We evaluated the effects of shoe heel height and pylon height on the loading rate on the contralateral limb. This helped develop software code that generated simulated data similar to experimental data. To test the effects of different algorithm versions, we first developed a program that generated simulated data. This simulated data is based on experimental pilot data from one participant with random noise added. By generating simulated data, we could pilot-test how well different human-in-the-loop optimization algorithms worked and determine the hyper-parameters. This methodology is similar to strategies used in previous studies (Felt et al., 2015; Ding et al., 2018).

2.1 Parameter-Sweep Case Study for Simulated Data Generation

As a preliminary step before conducting human-in-the-loop optimization, we collected data from one participant in which we tested all the possible combinations of shoe heel height and pylon height. Using this data, we plotted the relationship between shoe heel height and pylon height and their effects on contralateral limb loading (Supplementary Figure S1). Even though the goal of the algorithm was to reduce the loading rate of the contralateral limb, we used the data from both legs of the participants. Because of this, we were able to generate a greater variation of different types of simulated data that allowed us to develop, evaluate, and optimize our algorithm. We found that walking with different parameter combinations alters the loading rate on the contralateral limb. Additionally, the parameter sweep showed a minimum value or area, indicating an optimal parameter combination. Using this data, we could start developing the optimization algorithm.

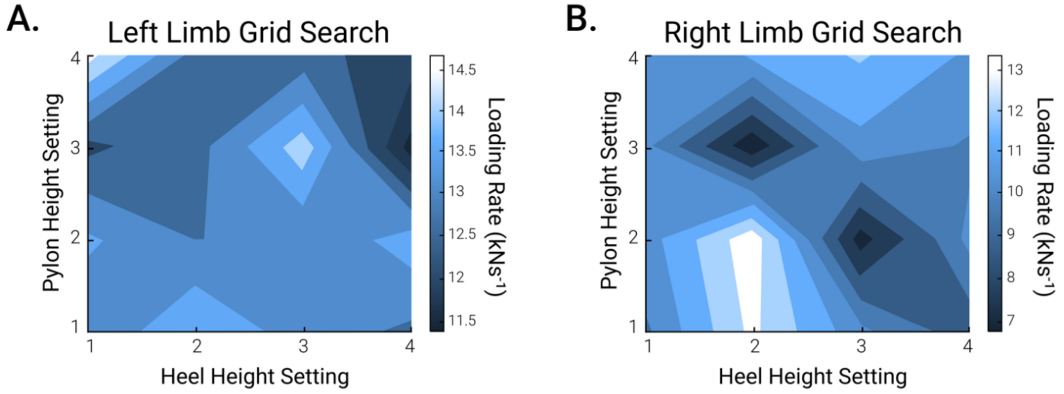

Supplementary Figure S1: Parameter sweep from preliminary case-study. The vertical axis shows different pylon height parameters with 1 being the lowest height and 4 being the highest height. The horizontal axis shows the different shoe heel height parameters with 1 being no height and 4 being the highest height. The bar on the right of the grid provides a color scale of the loading rate. The parameter-sweep includes all 16 combinations and helped provide a visual on which combination of shoe heel height and pylon height produced the lowest (dark blue) and the highest (white) loading rate.

2.2 Simulated Data Generation

From the pilot tests conducted, we developed a program that allowed us to generate simulated data with similar characteristics to the actual experimental data. The simulation program generated data based on a surface fitted through the pilot data with trial-to-trial variability. This variability is scaled to match the standard deviation of the difference between the fitted and actual data from the pilot test. We used this simulated data to test how well different human-in-the-loop optimization algorithms would work, similar to strategies used in previous studies (Felt et al., 2015; Ding et al., 2018). Additionally, we used this data to evaluate our human-in-the-loop optimization algorithm and fine-tune our hyper-parameters (α_0, A_0, and γ). We created a script that allowed us to generate a plot using a third-order polynomial fit based on the simulated data (Supplementary Figure S2). This allowed us to visualize the grid search of all the conditions. Additionally, we used this plot to validate the functionality of the human-in-the-loop optimization algorithm. This means that if there was a local minimum present on the plot, we had an idea of not only which direction the algorithm should go but where the algorithm should end up. Essentially, this visual representation was used to verify the algorithm's accuracy and fine-tune our hyper-*parameters (α_0, A_0, and γ).*

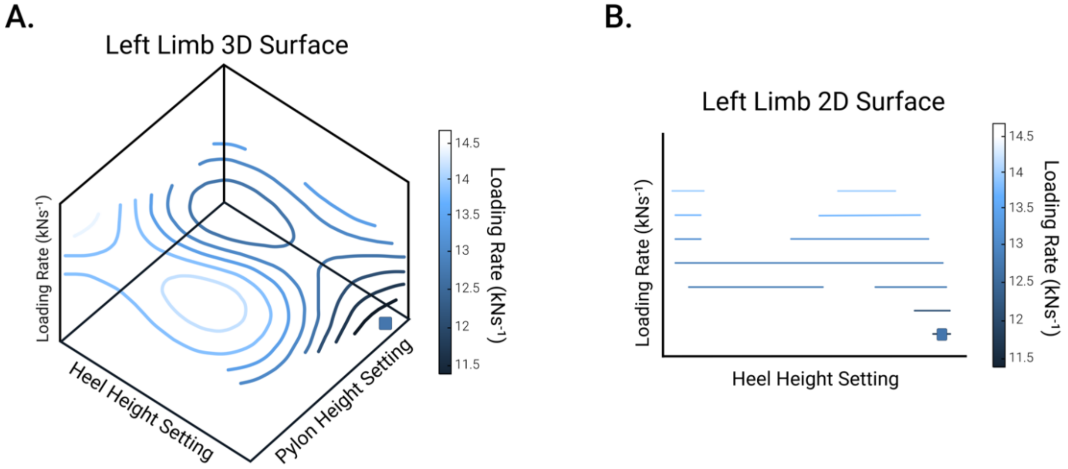

Supplementary Figure S2: Fitting the data. The human-in-the-loop optimization algorithm used a third-order polynomial to create a fit for the data. **(A) 3-Dimensional view.** the vertical axis shows the loading rate. The horizontal axis' show the shoe heel height and pylon height. The third-order polynomial fitted the data as a contour plot which guided the algorithm to the optimal combination (dark square). **(B) 2-Dimensional view.** the vertical axis shows the loading rate. The horizontal axis shows the shoe heel height. The third-order polynomial displayed the data as a hilly landscape where the lowest loading rate (dark blue) was displayed, similar to a valley.

We created a script that allowed us to generate simulated loading rate data according to the following function:

$$\mathbf{z_{Fit} = c_1x^2 + c_2x + c_3y^2 + c_4y + c_5xy + c_6 + c_7\eta} \tag{7}$$

where x, y, and z are shoe heel height, pylon height, and loading rate, respectively, c_1 to c_7 were optimized to fit the left leg loading rate data from the simulated pilot data, and η is random noise

between ± 0.5, and c_7 was chosen so that the variation of the simulated data around the surface determined by c_1 to c_7 mimics the variation of the measured pilot data.

3 Performance Comparison of Different Candidate Human-in-the-loop Optimization Algorithms

To select the human-in-the-loop algorithm for the study, we compared the performances of three candidate algorithms, including covariance matrix adaptation evolution strategy (CMA-ES), gradient descent, and successive parabolic optimization. After completing the study, we also briefly evaluated one additional candidate algorithm—Bayesian Optimization with Gaussian process—to investigate if it could have been a better alternative.

The successive parabolic optimization was the algorithm described in the study. The gradient descent algorithm is similar to the algorithm from the study but does not use parabolic optimization. The CMA-ES is based on the supplementary MATLAB code from Zhang et al. (Zhang et al., 2017). The Bayesian optimization algorithm employs the fitrgp function in MATLAB with a squared exponential kernel function to train the Gaussian process. Each algorithm was modified to work within the constraints of the current prosthesis optimization problem by limiting solutions to a 4x4 grid of possible combinations and constraining the tested conditions at each iteration to integer values only.

For each human-in-the-loop algorithm, we first tuned its hyperparameters, such as the scheduled gain parameters in gradient descent, the step size parameter (σ) in CMA-ES, or the number of initial points and the exploration-exploitation parameter (κ) in Bayesian optimization. We adjusted the hyperparameters by performing a CMA-ES optimization to search for hyperparameters that minimize a cost function based on combinations to convergence and a penalty term if the combinations to convergence is not defined. The optimization of the hyperparameters was restarted 20 times, after which we selected the median of the tuned settings for each hyperparameter to evaluate the performance of each algorithm.

After tuning the hyperparameters, we assessed the performance of each tuned algorithm based on combinations to convergence. We ran each algorithm 20 times with the same tuned hyperparameters but with slightly different simulated data using the random noise generation in the simulated data. We then evaluated the mean and standard deviation of the combinations to convergence (Supplementary Figure S3).

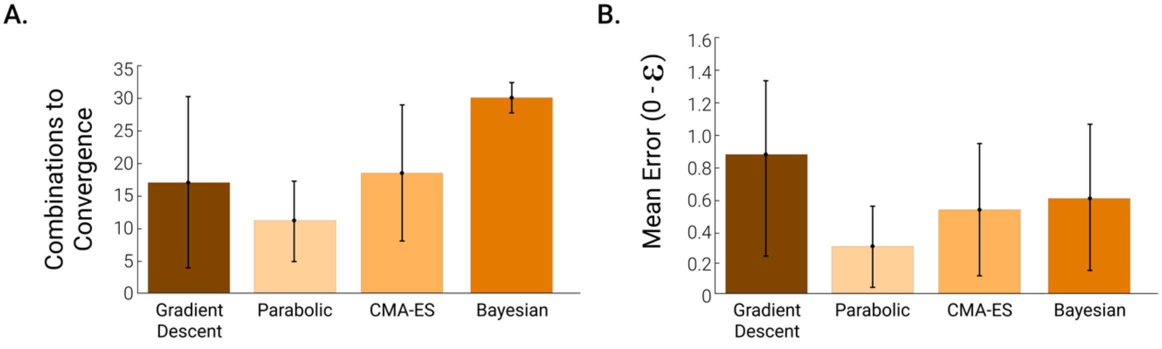

Supplementary Figure S3: Comparison of performance of different candidate human-in-the-loop optimization algorithms on simulated data. (A) mean ± SD of combinations to convergence of

20 simulated optimizations with a maximum of 32 combinations. If convergence was not achieved after 32 combinations, a score of 33 was given. **(B)** mean ± SD of error in the estimated optimum. The presented error is the mean of the error in the x and y direction (shoe heel height and pylon height, respectively).

The results of the simulated comparison of different algorithms suggest that successive parabolic optimization performed better than the other algorithms, which is surprising. The relatively lower effectiveness of the other algorithms may be due to the small resolution of the grid of possible combinations. Another possibility is how we evaluated algorithms based on convergence disfavors algorithms that tend to include combinations away from the optimum to avoid getting stuck in local minima. Finally, the parabolic optimization may have worked well because we used a paraboloid-like function to generate simulated data.

4 Quantifying Performance

Previous studies have used time-to-convergence as a performance metric for human-in-the-loop optimization algorithms (Felt et al., 2015; Zhang et al., 2017; Ding et al., 2018). We used a similar convergence metric to evaluate the performance of our algorithm (Supplementary Figure S4). An optimal combination was said to be achieved when prescribed combinations remained between the parameter setting one above and one below the estimated optimal parameter setting. In addition, the number of combinations it takes before combinations stay within the band was defined as 'combinations-to-convergence.'

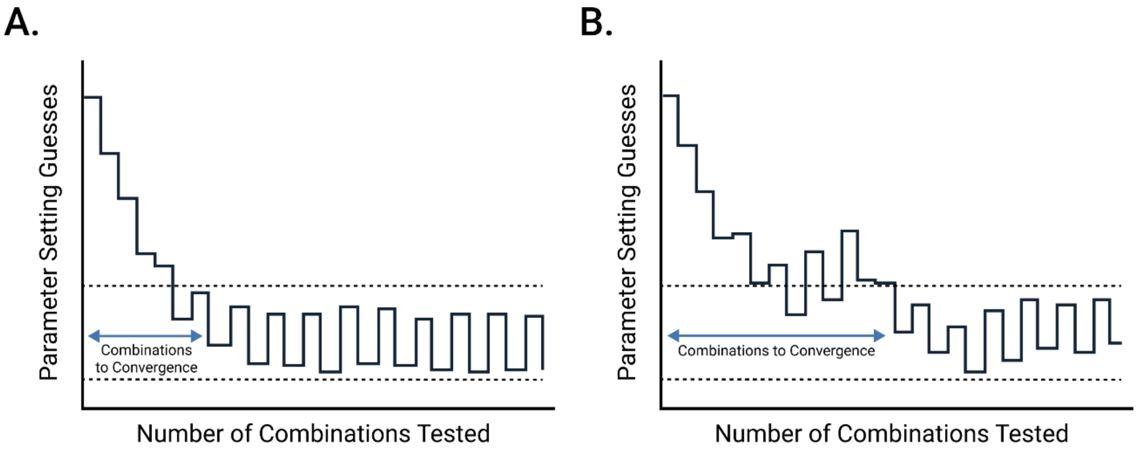

Supplementary Figure S4: Evaluation of performance using the convergence metric. The vertical axis shows the parameter setting guesses for either shoe heel height or pylon height, and the horizontal axis is the number of combinations tested. Ideally, the optimization algorithm should converge towards the optimal combination as quickly as possible (i.e., the minimal number of combinations to convergence) and as accurately as possible. **(A)** Example of a well-configured algorithm that finds the optimal parameter setting in a small amount of time and stays within the determined band **(B)** Example of a sub-optimal algorithm that takes a long time to find the optimal parameter setting.

5 Analyses of secondary biomechanical outcome variables

5.1 Per subject HIL Optimization

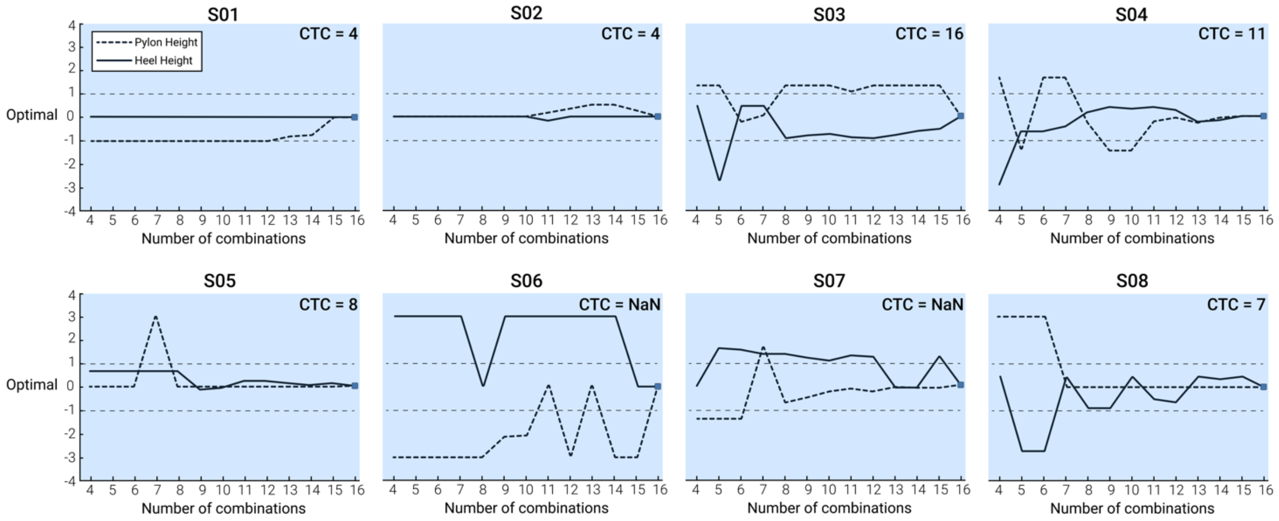

Supplementary Figure S5: HIL Optimization Plots. These are the patterns of each HIL optimization protocol for each participants. Shoe heel height and pylon height are represented as a solid and dashed line, respectively. 'CTC' refers to the corresponding combinations to convergence metric for each participant.

5.2 Per subject Sweep

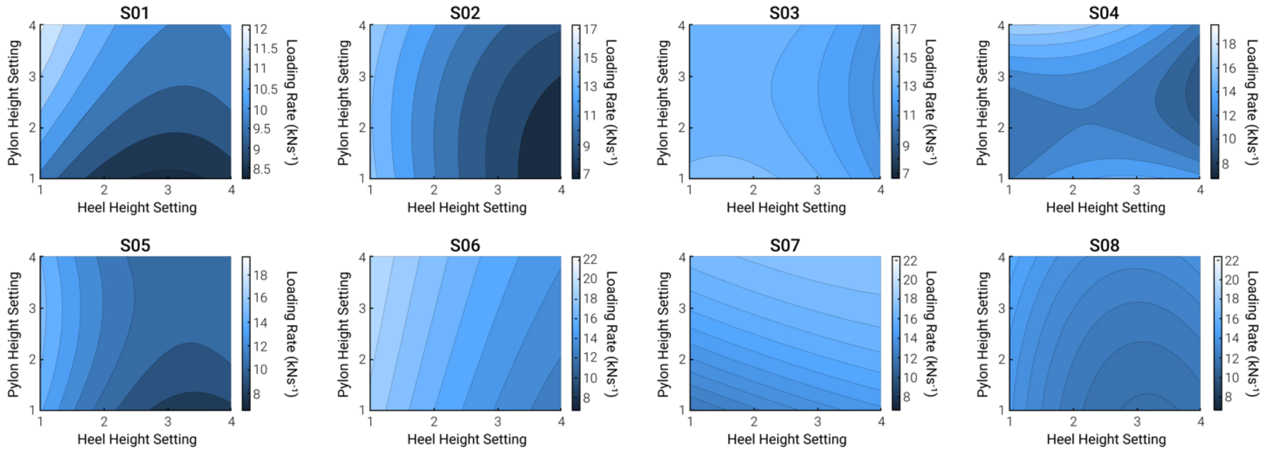

Supplementary Figure S6: Sweep Plots. We fit a second-order polynomial that was a function of shoe heel height and pylon height against the loading rate for each participant to determine the individual optimal combination.

5.3 Loading Rate Validation Comparison

For the participants who converged (n = 6), the average loading rate from the sweep optimum was 10.9 ± 1.3 kN s^{-1}. The average loading rate from the *HIL optimization* optimum was 10.4 ± 1.3 kN s^{-1}. The loading rate in the neutral combination setting (combination 1,3) was 15.3 ± 3.9 kN s^{-1}. There was no significant difference in the loading rate between the two optimal combinations (P = 0.062, Supplementary Figure S7). The *sweep* optimum and the *HIL optimization* optimum had a significantly lower loading rate than the neutral combination (P < 0.05).

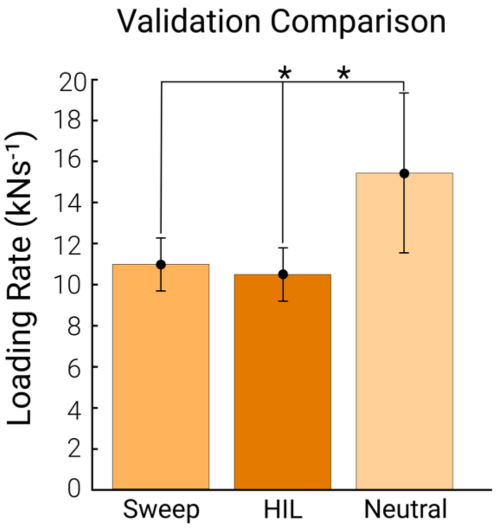

Supplementary Figure S7: Loading rate comparison. The average loading rate across participants who converged to an optimal combination from the *sweep,* the *HIL optimization* (HIL), and the neutral combination. The error bars represent the standard deviation across participants (n = 6).

5.4 Ground Reaction Force Profiles

In addition to evaluating the effects of the two optimization methods (*sweep* and *HIL optimization*) on our main objective parameter (vertical instantaneous loading rate), we also qualitatively evaluated the entire loading rate in this chapter. The purpose of this qualitative evaluation is to investigate how both optimization methods minimized loading rates. For example, does this happen by minimizing the entire ground traction force signal, or did a loading rate reduction happen at the expense of an increase in another metric (e.g., the peak)?

We plotted the ground-reaction force profile of each of the optimized combinations and the neutral combination to understand how the changes in loading rate are obtained by alterations in the ground-reaction force (Supplementary Figure S8). Although the *sweep* optimum and the *HIL optimization* optimum reduced the loading rate, these combinations produced a later but higher initial peak ground reaction force than the neutral combination.

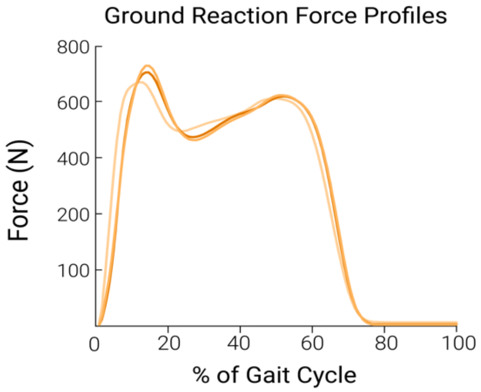

Supplementary Figure S8: The average ground reaction force profiles for the *sweep* optimum (orange), the *HIL optimization* optimum (dark orange), and the neutral combination (light orange) across all participants (n = 8).

5.5 Relative Limb Height Comparison

To understand how the changes in the pylon height and shoe heel height produce changes in loading rate, we also analyzed a measure of the functional leg length difference produced by changes in pylon height and shoe heel height. To approximate the produced relative limb height differences between conditions, we used a motion capture system (VICON Vero, Oxford Metrics, UK) and placed retro-reflective markers on the bony landmarks of the left and right anterior superior iliac crest of each participant.

We evaluated if there was a significant difference in relative limb height between the optimal from the two protocols (*sweep* and *HIL optimization)*, the neutral combination (combination 1,3), and walking without the device. For this purpose, we used one-way ANOVA with repeated measures. If there was a significant difference, we used a post-hoc paired t-test.

We used the z position of the left and right anterior superior iliac crest to determine if conditions resulted in a relative limb height difference due to a sideways pelvic tilt during standing. The height difference was calculated using the following equation:

$$\textbf{Relative Limb Height}_{\textbf{ASI}} = \textbf{Avg. Distance}_{\textbf{Left}} - \textbf{Avg. Distance}_{\textbf{Right}} \qquad \textbf{(8)}$$

where the Avg. Distance was calculated using the average z-position from the first 22 frames exported from motion capture, as these initial frames were consistently tracked across all participants. From the one-way ANOVA with repeated measures, there was a significant effect of the optimal parameter combinations on the relative limb height ($P < 0.05$, Supplementary Figure S9). The post hoc paired t-tests found no significant difference in limb height between the neutral combination and without the knee crutch ($P = 0.174$). The pelvis was significantly elevated on the side of the contralateral limb in the *HIL optimization* optimum compared to walking without the knee crutch ($P < 0.05$). Additionally, the pelvis was significantly elevated on the side of the contralateral limb leg in the *sweep* optimum than without the knee crutch ($P < 0.05$). The *sweep* and *HIL optimization* optimum had no significant difference in limb height ($P = 0.092$). There was no significant difference in relative limb height between the *sweep* optimum and the neutral combination and the *HIL optimization* optimum and the neutral combination ($P = 0.065$ and 0.228, respectively).

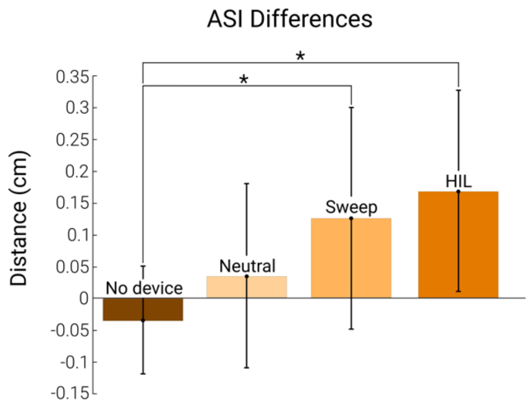

Supplementary Figure S9: Relative limb height differences. The average distance from the left to right anterior superior iliac crest across all participants for the following conditions: optimal combination determined by the *sweep* (sweep), the optimal combination determined by the *HIL optimization* (HIL), the neutral combination, and without the knee crutch. The error bars represent the standard deviation. The asterisks indicate significant differences (n = 8).

6 Variability Analyses

6.1 Effects of Shoe Heel Height and Pylon Height on Stride-to-Stride Variability

In addition to analyzing secondary biomechanical outcomes, we wanted to evaluate the stride-to-stride variability between parameter combinations. If parameters close to the optimum have greater variability in loading rate, this could result in a more variable performance of the HIL optimization algorithm. To perform this analysis, we first calculated the loading rate of individual strides (this is different from the analyses in the main study, which calculated the loading rate on the mean stride of one minute or longer). Next, we calculated the coefficient of variation between the strides for each parameter combination in the sweep protocol for each participant. To analyze how variable the loading rate was as a function of the parameter combinations, we fit a second-order polynomial that was a function of shoe heel height and pylon height against the coefficient of variation for each participant (Supplementary Figure S10).

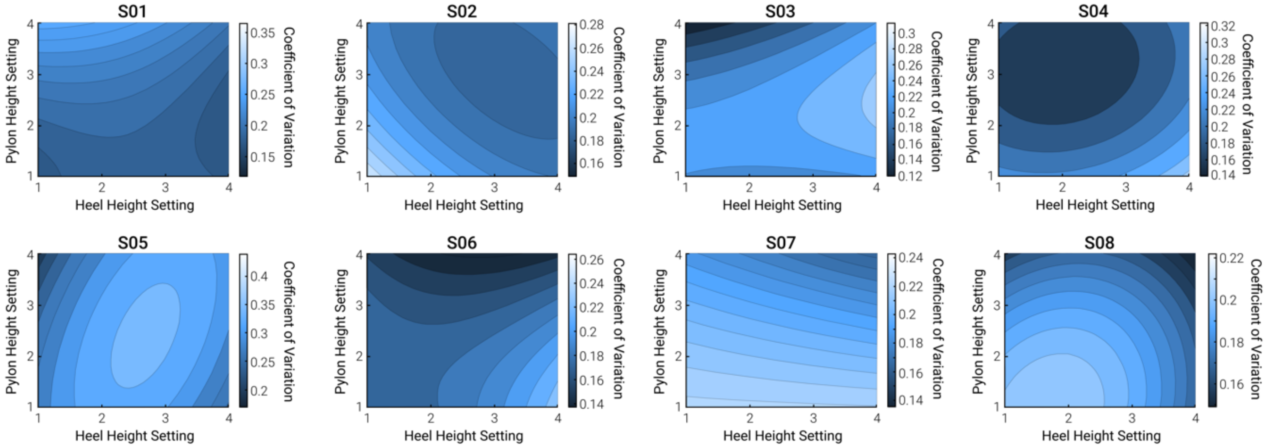

Supplementary Figure S10: Coefficient of variation plots. We used a second-order polynomial for each participant to evaluate the variation between parameter combinations.

To evaluate if there is an overall trend of effects of parameter combinations on stride-to-stride variability, we used the coefficient of variation from each participant from the sweep protocol to run a linear mixed-effect model. We used the following linear mixed-effect model (9) to study the effects of shoe heel height and pylon height on the coefficient of variation across parameter combinations:

$$\mathbf{z_{Fit} = c_1x^2 + c_2x + c_3y^2 + c_4y + c_5} \qquad \textbf{(9)}$$

where x, y, and z are shoe heel height, pylon height, and coefficient of variation, respectively, terms c_1 to c_4 are the coefficients for each independent parameter setting, and c_5 is the constant intercept term. We found no statistical significance for each of the terms (P-values were 0.225, 0.128, 0.823, and 0.743

for shoe heel height, the square of shoe heel height, pylon height, and the square of pylon height, respectively; Supplementary Figure S11). It appears that there is a trend that lower pylon heights result in smaller stride-to-stride variability. However, findings from the statistical analysis suggest that the step-to-step variability in loading rate did not change in a consistent way as a function of shoe heel height and pylon height (e.g., walking with lower heel heights was not less variable).

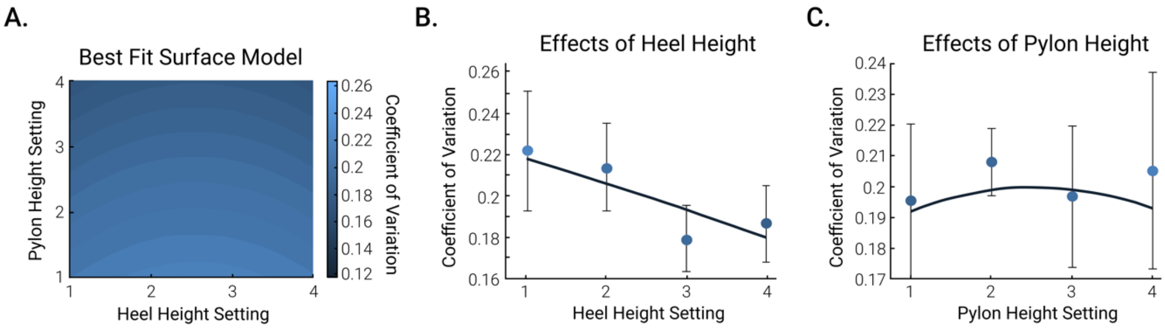

Supplementary Figure S11: Linear mixed-effect model. We used a 2nd order polynomial to analyze the effect of shoe heel height and pylon height on the stride-to-stride loading rate variability. **(A) The surface plot of the linear best-fit model**. The pylon height setting is on the vertical axis, and the shoe heel height setting is on the horizontal. The color bar represents the coefficient of variation, where light blue is the highest and dark blue is the lowest. **(B, C) 2-Dimensional plot.** The effect of shoe heel height (B) and pylon height (C) on the step-to-step variability. This 2-dimensional plot was taken from the middle point of pylon height and shoe heel height from (A), the mean of conditions 2 and 3. The circles and error bars in (B) represent the mean ± standard deviation of all pylon heights at each shoe heel height setting. The circles and error bars in (C) represent the mean ± standard deviation of all shoe heel heights at each pylon height setting (n = 8).

Since we used the neutral parameter combination in the familiarization session (session 1), we also wanted to compare the step-to-step variability in the neutral parameter combination to all other parameter combinations. We calculated the coefficient of variation for the neutral parameter combination (Supplementary Figure S11) and calculated the mean coefficient of variation across all the other combinations. Using a paired t-test, we found that the neutral parameter combination had a significantly lower coefficient of variation than all other parameter combinations ($P < 0.05$, $n = 8$). This could suggest that participants were more habituated to the neutral condition.

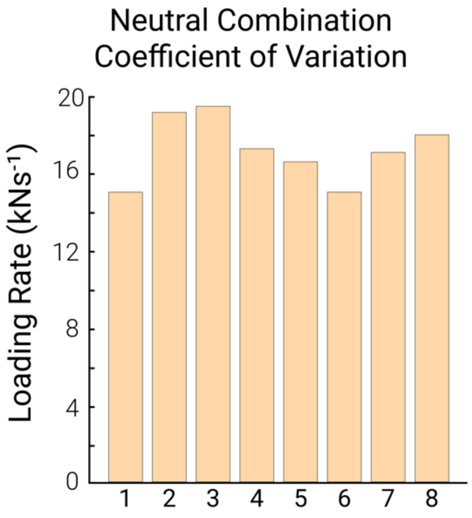

Supplementary Figure S12: Coefficient of variation for the neutral parameter combinations. We calculated the coefficient of variation for the neutral parameter combination for each participant.

6.2 Analysis of Trial-to-Trial Repeatability

We did not purposively repeat conditions in order to perform a repeatability analysis; however, within the HIL optimization protocol, certain combinations were repeated. In this chapter, we analyzed these repeated conditions to understand whether the fact that certain repeated combinations produced different results could explain HIL optimization performance.

First, we identified for each participant which combinations were repeated. Each participant had at least one combination that occurred twice. Next, we calculated the variation coefficient between those repeated combinations. On average, the coefficient of variation of the repeated combinations in the HIL optimization protocol is 0.086 ± 0.046. A coefficient of variation below 0.10 is considered good.

As another point of reference, we compared the coefficient of variation of these repeated combinations to the variation among all the combinations in the HIL optimization protocol. Interestingly when we calculate the mean and standard deviation of the coefficient of variation of all HIL combinations of each participant, this only amounts to 0.146 ± 0.063. In summary, it appears that the variation in loading rates obtained by changing combinations is only slightly over 50% larger than the variation due to the repeatability of the measurements. This relatively high noise-to-signal ratio could partially explain the limited HIL optimization performance.

To further investigate this finding, we calculated if there is a correlation between the ratio of the coefficient of variation of the repeated combinations versus the coefficient of variation of all combinations and the HIL optimization performance expressed as combinations to convergence (Supplementary Figure. S13). This analysis suggests that participants with a more favorable noise-to-signal ratio had a smaller combinations to convergence and vice versa. This finding should be nuanced, however, since one of the two participants who did not show convergence did not follow this trend (this participant seemed to have had good repeatability compared to the coefficient of variation of all the combinations in the HIL protocol).

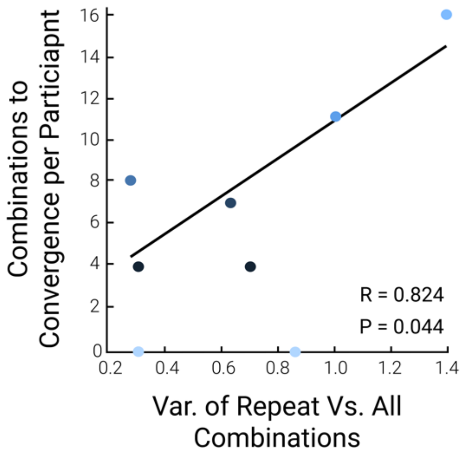

Supplementary Figure S13: Relationship between a noise-to-signal ratio metric and time-to-convergence. The horizontal axis shows the ratio between the variability between combinations repeated during the HIL optimization protocol divided by the variability of all combinations in the HIL optimization protocol. The vertical axis shows combinations to convergence. Each circle represents one participant with the colors corresponding to the combinations to convergence value (light blue = no convergence, dark blue = 16 combinations to convergence). The black line shows a linear trend in data from participants with a defined combinations to convergence (noise-to-signal ratio from participants who did not have a defined combinations to convergence are also shown on the X-axis but not included in the trendline calculation).

6.3 The Effect of Trial Duration on Loading Rate Error

We evaluated the required walking duration for accurately determining a loading rate based on a supplementary analysis of the validation trials of the sweep optimum. For each participant, we determined the loading rate from an average trial from all three minutes of this trial. 3 minutes is a relatively long duration that even exceeds the duration required to estimate very noisy parameters such as metabolic cost (Selinger and Donelan, 2014; Zhang et al., 2017). Therefore, we assumed that 3 minutes should definitely be long enough to determine a loading rate accurately, and we defined the 3-minute loading rate as the "true" loading rate of the validation trial.

Next, we determined loading rates from different lengths of subsamples going from 1 stride all the way to the maximum possible subsample duration of 3 minutes. Finally, we calculated the absolute error between the loading rates of the different subsample lengths and the true loading rate and plotted this versus the subsample duration (Supplementary Figure S14). This analysis shows that short recording durations have high variability between strides resulting in large errors, and longer loading rates have smaller errors. Plotting the mean trend from all participants shows that recording for 60s is sufficient to reduce the error below 5%. It should be noted that this analysis was done after the completion of the main experiment. The trial duration for the main experiment was chosen based on informal pilot testing rather than the present comprehensive analysis.

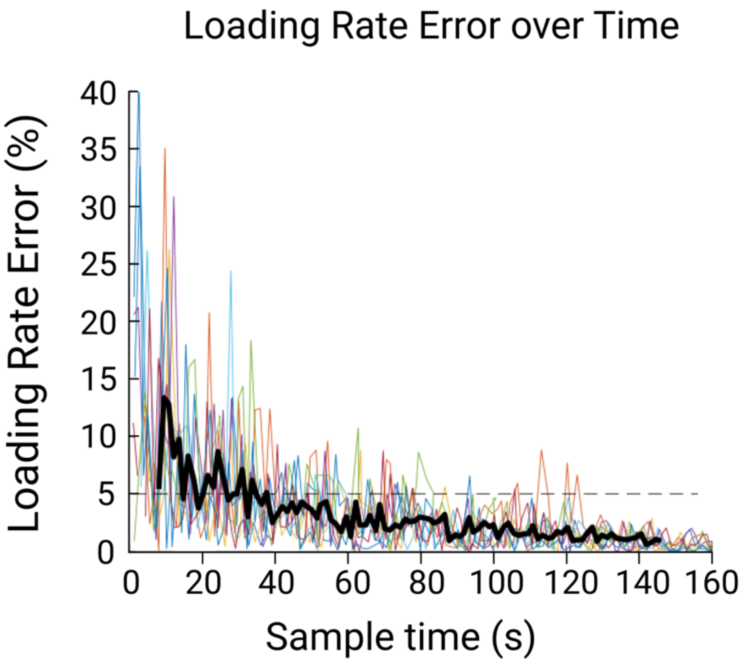

Supplementary Figure S14: Analysis of required sample duration. Lines show absolute error in loading rate as a function of sampling duration. Colored lines represent different participants. The thick black line is the mean trend from all participants.
